# Harnessing the power of the grassroots to conduct public health research in sub-Saharan Africa: a case study from western Kenya in the adaptation of community-based participatory research (CBPR) approaches

**DOI:** 10.1186/1471-2458-13-91

**Published:** 2013-01-31

**Authors:** Allan Kamanda, Lonnie Embleton, David Ayuku, Lukoye Atwoli, Peter Gisore, Samuel Ayaya, Rachel Vreeman, Paula Braitstein

**Affiliations:** 1 Moi Teaching and Referral Hospital, Eldoret, Kenya; 2 Dalla Lana School of Public Health, University of Toronto, Toronto, Canada; 3 Department of Behavioral Sciences, College of Health Sciences, School of Medicine, Moi University, Eldoret, Kenya; 4 Department of Medicine, Moi University, College of Health Sciences, School of Medicine, Eldoret, Kenya; 5 Department of Pediatrics, Moi University, College of Health Sciences, School of Medicine, Eldoret, Kenya; 6 Department of Children’s Health Services Research, Indiana University, School of Medicine, Indianapolis, USA; 7 Department of Medicine, Indiana University, School of Medicine, Indianapolis, USA; 8 Regenstrief Institute, Inc, Indianapolis, USA

**Keywords:** Community-based participatory research, Sub-Saharan Africa, Orphaned and separated children

## Abstract

**Background:**

Community-based participatory research (CBPR) is a collaborative approach to research that involves the equitable participation of those affected by an issue. As the field of global public health grows, the potential of CBPR to build capacity and to engage communities in identification of problems and development and implementation of solutions in sub-Saharan Africa has yet to be fully tapped. The Orphaned and Separated Children’s Assessments Related to their Health and Well-Being (OSCAR) project is a longitudinal cohort of orphaned and non-orphaned children in Kenya. This paper will describe how CBPR approaches and principles can be incorporated and adapted into the study design and methods of a longitudinal epidemiological study in sub-Saharan Africa using this project as an example.

**Methods:**

The CBPR framework we used involves problem identification, feasibility and planning; implementation; and evaluation and dissemination. This case study will describe how we have engaged the community and adapted CBPR methods to OSCAR’s Health and Well-being Project’s corresponding to this framework in four phases: 1) community engagement, 2) sampling and recruitment, 3) retention, validation, and follow-up, and 4) analysis, interpretation and dissemination.

**Results:**

To date the study has enrolled 3130 orphaned and separated children, including children living in institutional environments, those living in extended family or other households in the community, and street-involved children and youth. Community engagement and participation was integral in refining the study design and identifying research questions that were impacting the community. Through the participation of village Chiefs and elders we were able to successfully identify eligible households and randomize the selection of participants. The on-going contribution of the community in the research process has been vital to participant retention and data validation while ensuring cultural and community relevance and equity in the research agenda.

**Conclusion:**

CBPR methods have the ability to enable and strengthen epidemiological and public health research in sub-Saharan Africa within the social, political, economic and cultural contexts of the diverse communities on the continent. This project demonstrates that adaptation of these methods is crucial to the successful implementation of a community-based project involving a highly vulnerable population.

## Background

Community-based participatory research (CBPR) is a collaborative approach to research that involves the equitable participation of those affected by an issue in the research process. This approach enhances the understanding of a problem within the social, political, economic and cultural context of the community with the goal of taking action to improve the health and well-being of community members [1-5]. This research methodology is being increasingly adapted as a framework for conducting epidemiological research [3]. It provides a platform for local capacity building, systems development, knowledge transfer and exchange, and individual and community empowerment [1-3]. The CBPR process is characterized by forming community partnerships and consultations, and entering into meaningful community engagement from the development of the research agenda through to the interpretation, dissemination and application of findings [1-3,6]. CBPR can employ both quantitative and qualitative research designs and approaches [2,7]. It has been applied across a broad spectrum of settings, and is particularly useful for engaging marginalized and hard-to-reach populations [1,7].

The majority of the literature on and about CBPR is from Canada and the United States, where the concepts and methodologies of CBPR have been the most extensively developed [5]. However, as the field of global health grows, and as researchers from around the world begin to focus more attention on urgent public health issues affecting sub-Saharan Africa and other resource-constrained settings, the potential of CBPR to build community capacity and to engage communities in identification of problems and development and implementation of solutions has yet to be fully tapped. Sub-Saharan Africa is composed of a diversity of cultures and communities with strong community networks and civil structures in both rural and rapidly urbanizing communities [8], yet the continent is faced with a high burden of disease and many public health issues [9,10]. Harnessing the power of CBPR can have a significant impact on improving the population’s health and well-being in the region and empowering communities to take action by addressing important public health issues [7,11].

**Figure 1:**
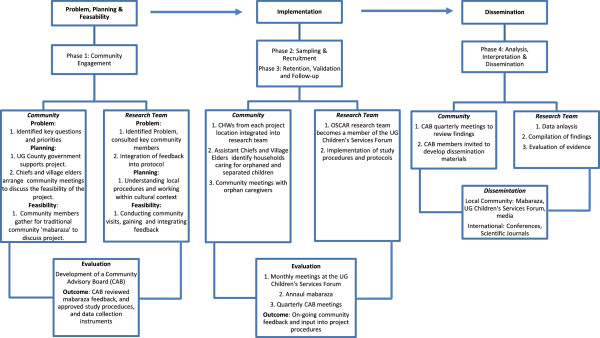
CBPR framework.

The Orphaned and Separated Children’s Assessments Related to their Health and Well-Being (OSCAR) project is a 5-year longitudinal cohort study evaluating the effects of different care environments on the physical and mental health outcomes of orphaned and separated children in western Kenya. This paper will describe how a CBPR framework (Figure 1) that addresses problem identification, planning and feasibility; implementation; and evaluation and dissemination can be adapted and incorporated into the study design and methods of a longitudinal epidemiological study in sub-Saharan Africa using the “OSCAR’s Health and Well-being Project” as a case study. We will illustrate how a research project of this nature would not be feasible without full community engagement and support and that community participation is integral to ensure the research design and study methodology are in alignment with the social, cultural and political context of the country. We describe our application of the CBPR framework in four phases: community engagement; sampling and recruitment; retention, validation and follow-up; and analysis, interpretation, and dissemination.

### Community-based participatory research in sub-Saharan Africa

Sub-Saharan Africa faces a multitude of challenges and a disproportionate burden of disease, with many public health issues at the forefront that require solutions. Only 31% of the population has access to improved sanitation facilities, and 40% lack an improved drinking water source [12]. Approximately 45% of the world’s under-five mortality occurs in the Africa region, with the majority of deaths due to diarrheal diseases, pneumonia, malaria and HIV [9]. The continent carries 68% of the global HIV burden [13] and has the highest Disability Adjusted Life Year rates in the world [9]. These public health challenges cannot be addressed without considering the social, cultural, political and economic factors influencing health outcomes at the macro (continent-wide) and micro (local community-level) level. The large majority of premature deaths in sub-Saharan Africa are preventable by relatively simple and inexpensive interventions, which can be implemented at the community-level [14].

Community participation increases the likelihood that the project will be culturally and educationally appropriate; its format and content will better fit the cultural systems of the community [2]. Additionally, community participation increases the sustainability of an intervention and the likelihood of its long-term success and influence on public health policy [4]. This existing evidence thus suggests that adapting these principles of community participation to research in sub-Saharan Africa will assist in effectively addressing the root causes of public health problems, and finding sustainable, culturally appropriate solutions.

### Orphans and separated children in sub-Saharan Africa

Communities in sub-Saharan Africa have been faced with the growing challenge of providing care to orphaned and separated children. An orphaned child is defined by UNICEF as having as having lost one (single orphan) or both parents (double orphan) [15]. Separated children are virtually orphaned due to the absence of one or both of their biological parents [16,17]. The total number of orphans from all causes has been decreasing in Asia, Latin America, and the Caribbean since 1990. However, the number of orphans in sub-Saharan Africa has increased during this same time by more than 50%, largely due to the AIDS pandemic [15]. Traditionally, orphaned or separated children would be absorbed by the extended family, and it is estimated that 90% of double orphans are still cared for by their extended family [15,18]. Family care for orphans is usually preferred by children and families, and highly regarded by policymakers [19-21]. However, in communities where the AIDS epidemic has advanced, there may be fewer available caregivers and a growing number of overwhelmed and dissolving households. The sheer numbers of children requiring care and support, layered on top of pre-existing poverty, can prevent some families from meeting traditional care-taking expectations and responsibilities [18,22-26]. This puts additional strain on communities to absorb orphaned or separated children, and may leave children with inadequate care, particularly in settings of abject poverty. With a growing number of orphans in sub-Saharan Africa, orphans’ health and well-being is a public health priority that requires attention in order to ensure the future stability and development in the region.

### OSCAR’s Health and Well-Being Project

This project is a 5-year community-based longitudinal cohort study evaluating the effects of different care environments on the physical and mental health outcomes of orphaned and separated children. The study began enrolling participants in July 2010. The project follows a cohort of orphans and separated children from communities within 8 locations, representing 300 households, 20 Charitable Children’s Institutions (CCI’s) and 7 community-based organizations, in the Uasin Gishu (UG) County of western Kenya. To date the study has enrolled 3130 participants in total with 1526 children from CCI’s, 1504 from households, and 100 street-involved children and youth. The study has 3 primary specific aims: 1) to characterize models of care for children who are orphaned or separated (i.e. actually or virtually orphaned children); 2) to investigate the effect of care environment on child and household socioeconomic indicators; 3) to measure the effect of care environment on the physical and mental health of the resident orphaned and separated children. The project utilizes standardized site assessments, annual medical examinations and psychosocial assessments to meet these aims.

UG County is one of the 47 counties of Kenya, located in the Rift Valley Province. In 2010, UG County had approximately, 894 179 individuals from 202 291 households, of whom 41.5% are aged 14 years or less [27]. The majority of the UG County population (61.4%) resides in rural settings [28] in comparison to 67.7% of the population in the rest of Kenya and 77.3% in East Africa [8]. Approximately 51.3% of the population in UG County live below the Kenyan poverty line (1,562 KES pp/month ~ 18.75 USD) [28]. The city of Eldoret is the County’s capital, administrative and commercial center. Eldoret has a total population of 289 389 and is currently, the 5th largest city in the country. It is home to Moi University (including Kenya’s 2^nd^ medical school), Moi Teaching and Referral Hospital, and the USAID-AMPATH (Academic Model Providing Access to Healthcare) Partnership [29,30], a USAID-PEPFAR funded organization that is actively providing HIV care and treatment to approximately 75,000 HIV-infected adults and children in western Kenya. AMPATH provides access to free antiretroviral treatment (ART), as well as comprehensive nutrition services, psychosocial support, and economic development opportunities. AMPATH has a highly functioning research network with shared North American and Kenyan leadership (http://research.ampath.or.ke/).

### Human subjects protection

This study was approved by the Moi University College of Health Sciences and Moi Teaching and Referral Hospital Institutional Research and Ethics Committee and the Indiana University Institutional Review Board. Informed consent was provided by the head of household, Director of CCI, and in the case of the street youth, by the District Children’s Officer (DCO). Individual written assent was provided by each child aged 7 years and above. Fingerprints were used for both children and guardians who were unable to sign or write their name.

## Problem identification, feasibility and planning

### Phase 1: community engagement

As the team considered various approaches and primary research questions, key opinion leaders in the community were engaged. Several CCI’s were visited and the team worked closely with the DCO to develop the project and ensure that it was addressing the community’s health concerns in relation to orphaned and separated children. CCI directors consistently raised the concern that the Government of Kenya has proposed closures of CCI’s due to the belief that children are ‘better off’ in the community than in institutions. This concern formed the underlying OSCAR project hypothesis that children living in CCI’s will have improved health and well-being outcomes compared to children in households in the community. Additionally, one of the CCI directors wanted to know whether CCI’s that are structured into smaller ‘family’ units of 15–20 children had better outcomes among their children compared to ‘dormitory’ style CCI’s. The DCO was explicit that he wished to conduct an evaluation of the government cash transfer program to determine its effectiveness in improving the health and well-being of orphaned children. As a result, the recruitment and randomization strategy for households in the community was planned and conducted in such a way as to be able to compare households receiving cash transfers to households in the same and different locations not receiving cash transfers. Both of these questions were integrated into the protocol submitted for funding as a result of the participation of key figures in the community to identify issues of importance.

#### Gaining community entry

**Figure 2:**
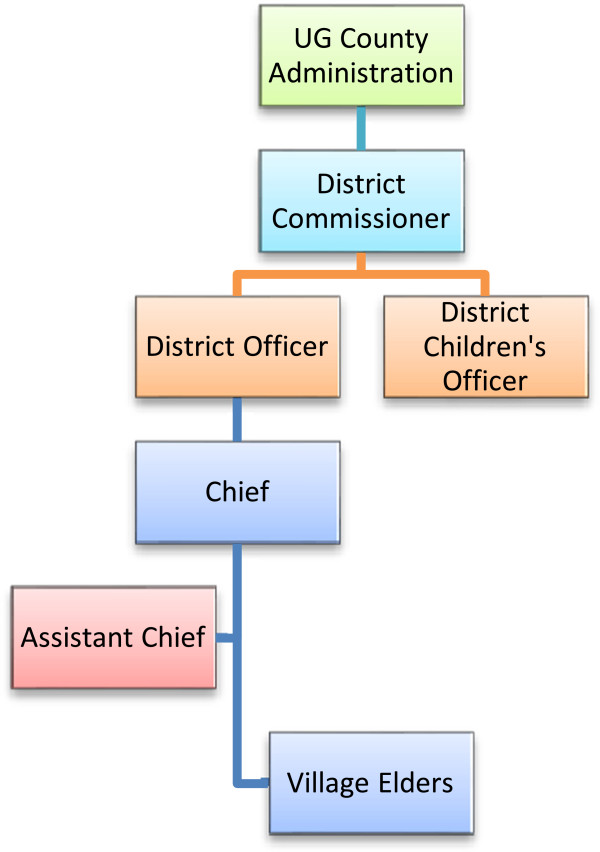
Administrative structure in Uasin Gishu County, Kenya.

The complex cultural, community and administrative systems (Figure 2) present in western Kenya required the project team to gain an in-depth understanding of these systems and follow local procedures for accessing the communities and carrying out research. The project team developed a plan that commenced by introducing the OSCAR’s Health and Well-being Project to the UG County’s District Administration, in order to gain support and facilitate community entry.

The DCO handles all children’s affairs in the county’s District Administration and is based in the county’s headquarters. The DCO was the first point of contact for the project in the government administration and was instrumental in introducing the project to the District Commissioners, who prior to the application for funding in turn provided the project a letter of authorization to carry out the research in the county. Authorization from the District Commissioners ensured the project was following the traditional administrative hierarchy and allowed the project to arrange meetings with the District Officers, as well as with Chiefs, Assistant Chiefs, Village Elders and opinion leaders in their respective Divisions.

The goal of these meetings was to explain the project and seek the support and involvement of the Chiefs, Village Elders, and community residents in eight locations. Six of these locations (three rural and three urban) were selected based on their receipt of a government cash-transfer subsidy for impoverished households caring for orphans. The two other locations (one rural and one urban) were selected purposively in neighbouring locations that did not receive the subsidy.

In order to facilitate on-going participation of community residents once funding was received, the project sought to hire Community Health Workers (CHW’s). CHW’s form the backbone of *Kenya’s Essential Package for Primary Health* care. Typically CHW’s have completed secondary school and are engaged to work in the community where they live. For this project, individual hiring processes were conducted in each of the locations where the project planned to work. Each application was required to be accompanied by a recommendation letter from the Chief of that community attesting to the individual’s residence in the community. During the interview process, CHW’s were challenged to describe key aspects of their community (e.g. names of Village Elders, names of sub-Locations, etc.) to ensure their knowledge of the community where they would be working.

### Conducting community visits

The project team chose to meet with the community through a series of *mabaraza* due to the cultural and traditional significance of the *baraza* (singular form of *mabaraza*) in East Africa. The *baraza* is a traditional form of community assembly in East Africa, and in Kenya they are held as official public gatherings with Chiefs and sub-Chiefs [31]. Village Elders in collaboration with the project’s CHW’s mobilized concerned community residents (i.e. those caring for orphaned and separated children, including sibling-headed households) to attend the *mabaraza*. Venues for the *mabaraza* ranged from the Chief’s camp, or church compounds to divisional headquarter grounds.

The project team composed of the investigators, project manager, clinic staff (doctor, nurse, social worker, outreach worker, clinical psychologist), and CHW’s attended the *mabaraza*. The meetings were conducted in Swahili which is Kenya’s national language. In addition, *mabaraza* in two rural locations also used translations into the local vernacular by the CHW’s and project social worker. During the *mabaraza*, the project was introduced and community members participated in detailed discussions. Community members asked various questions and sought clarifications about the project and these were addressed by the project team. The care of orphans and their physical and mental health was an important issue for residents in all the communities. Community members were invited to raise questions or issues that they had about the care of orphaned and separated children which the project could attempt to address. No substantive suggestions were made that would fit within the project focus on orphans (numerous suggestions were made about including disabled children irrespective of their orphan status, and of conducting a study of the elderly). Thereafter, permission was sought from the community whether they were in acceptance of the project goals and activities and if they would support it. The project team had to be sure that the community residents were in agreement with the project aims were and would get the full and continuous support of the community over the cohort’s five year span. The Chief would request one or two community members to summarize to the audience what he or she understood about the research project and this would be confirmed or corrected by other community members. This demonstrated community member comprehension and allowed the OSCAR project team to correct or elaborate on issues that weren’t fully understood. The project got overwhelming support from the community in the *mabaraza* and this was confirmed by answering back yes or by raising the right hand up.

### Community Advisory Board (CAB)

To ensure the community was able to work directly with the project team, the project set up a CAB whose members include: Village Elders, Assistant Chiefs, Chiefs, opinion leaders, representatives of CCI’s, DCO’s, and representatives of the orphaned children in UG County, other relevant stakeholders and the project co-investigators. Prior to commencing the implementation of the study, the CAB reviewed all information collected from community *mabaraza*, study procedures, and data collection instruments. In partnership with the project investigators, the CAB assisted in finalizing the assessment and data collection instruments to ensure cultural relevance as well as scientific validity of the resulting data. The CAB played an integral role in allowing the community to participate in developing the research agenda, implementing the project and assisting with on-going community activities.

## Implementation

### Phase 2: sampling and recruitment

#### Charitable Children’s institutions (CCIs)

Under the Kenyan Children Act (2001), orphanages and other institutions serving orphans are called CCI’s (i.e. children’s homes) if they are able to accommodate ≥ 20 children [32]. All such institutions being subject to the Kenyan Children Act (2001), located within UG county boundaries, were eligible for participation in and recruitment in the study. The UG County Children’s Department maintains a list of registered and unregistered institutions, and has monthly meetings with them in the UG Children’s Services Forum. Two methods were used to identify and recruit CCI’s to participate in the project. First the project utilized the lists of registered CCI’s maintained by the UG Children’s Department and contacted them with a formal letter of introduction from the DCO. Secondly, snowball sampling techniques were used with community members and other stakeholders to identify and contact non-registered CCI’s. The OSCAR project became a member of the UG Children’s Services Forum and was given the opportunity to discuss the research project with forum members. Support was sought from the forum members and the project hoped to identify and sample all eligible CCI’s. The CCI’s were instrumental in identifying names and locations of other CCI’s to the project that could be approached and introduced to the project. In total, there were 21 eligible CCI’s identified in the UG County through the two strategies that the project wanted to recruit. For those not able to attend the Forum meeting, we arranged individual meetings with them and/or their Boards of Directors to discuss the study. Of 21 identified eligible CCI’s in the UG County that were contacted, 20 agreed to participate and one declined. The project arranged appointments to visit the 20 CCI’s that agreed to participate to facilitate enrolment and assessments of children on their premises.

### Community household sampling

**Figure 3:**
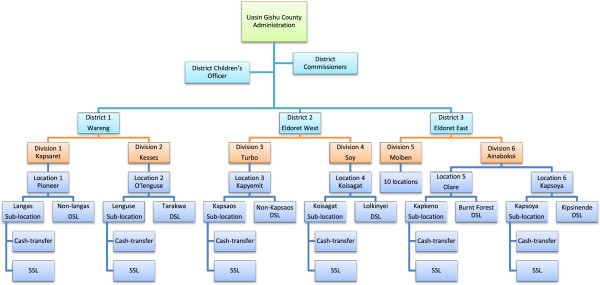
Sampling locations within Uasin Gishu County, Kenya.

The project aimed to randomly sample 300 households within eight locations representing families caring for orphaned and separated children in the UG County. In order to obtain a representative sample of households caring for orphans in UG County, the project utilized three sampling arms: cash-transfer (CT) households, non-cash transfer households from the same sub-Location (SSL), and non-cash transfer households from a different sub-Location (DSL) (Figure 3). Sub-Locations are administrative boundaries within locations and are headed by an Assistant Chief (Figure 2/Figure 3). 100 households were required from each category and weighted by Location to reflect the number of households required per location based on the population, to ensure appropriate distribution.

Assistant Chiefs and Village Elders drew up lists of all the households in their villages and sub-Locations caring for orphaned and/or separated children. The lists contained the names of the head of household, their national ID number where available, telephone number where available, the village in which they live, the number of children in the household, and the number of orphaned children in the household. These lists became the sampling frame for the random selection of SSL and DSL households to invite as per the sampling strategy just described.

The DCO oversees the government CT program and provided the study lists of households receiving the government subsidy in each location. These lists were used for simple random sampling for the CT households. CHW’s facilitated community meetings in each of the sub-Locations with the orphan caregivers. During these meetings, the project team explained the process of identifying and sampling eligible households in collaboration with the DCO, Chiefs, Assistant Chiefs and Village Elders. This was important as the project wanted to ensure transparency in its operations, particularly in the random sampling for recruitment into the project.

### Community household recruitment

Project CHW’s were required to identify randomly sampled households and confirm their eligibility in the eight Locations. To facilitate an introductory visit, CHW’s were accompanied by Village Elders at each Location. Village Elders are highly respected in the community, familiar with the households within their villages and have earned the trust of the community over years. The participation of the Village Elders played a vital role in establishing a relationship between the CHW’s and the households.

In subsequent visits, the CHW’s invited the households to participate in the research project. Households interested in participating were given appointment dates to visit the project clinic for enrolment and to participate in the consenting and assenting process with the project social worker before children received their annual assessments. Over time, the CHW’s have established a good rapport and trust with the communities and have played an essential role in facilitating enrolment and ensuring the on-going collaboration between the research project and the community.

### Phase 3: retention, validation, and follow-up

The CHW’s are an instrumental component of the project’s successful implementation. They are required to follow-up with each household at least quarterly. They monitor the households in their Locations for changes among the children or the guardians, moves within or outside of the Location, deaths (child or guardian), or any other issues affecting the on-going participation of an individual child or an entire household. For example, if there is a change of guardian (e.g. if the guardian who provided the initial informed consent dies), the CHW’s alert the study management and facilitate updating the consent on file. They pay close attention to whether there are new children in the household, whether children previously there have left, and whether any children have died. If and when there are changes in the household, the CHW for that Location is required to confirm the information either directly or in communication with Village Elders. CHW’s are also responsible for carrying out household assessments in the field. In this manner data collected on household indicators, including material assets, number of beds, number of children in the household, etc. are more reliable as they are collected on site by a local community resident. Data collected are validated through random household audits by study management throughout the year.

As a result, although children are formally assessed annually by the study, there is constant communication with, and monitoring of, enrolled households in the community throughout the year. The CHW’s are the critical on-going link between the study team and the community and as residents are able to provide continuous feedback from their respective communities to the project team.

## Evaluation and dissemination

### Phase 4: analysis, interpretation & dissemination

Analysis, interpretation and dissemination of the results of this project are on-going. The project is currently in its third year and will be carrying out annual assessments on enrolled children for at least 5 years. The CAB is playing an on-going and significant role in reviewing and evaluating the study’s data. The CAB aims to meet quarterly with the research team to review preliminary findings, discuss interpretation within the local context and determine appropriate dissemination strategies. As this research is on-going, the project is still in the process of compiling, and analyzing data. The CAB hopes to engage in advocacy, both directly with government officials and indirectly through public media, highlighting pertinent issues affecting the health and well-being of orphaned and separated children, including lobbying policy-makers in the Kenyan government, UNICEF, USAID, etc. and making policy recommendations based on the project’s findings.

Additionally, the project directly disseminates information semi-annually to the UG Children’s Services Forum and attends their monthly meetings. The project provides monthly updates to the DCO and other policymakers involved on issues affecting children. We continue to interact, collaborate, and disseminate information directly to the community through the CHW’s where we gain invaluable feedback and ensure the on-going cultural and community relevance. Presentations of the data in lay format through *mabaraza* are presently in the planning stages.

## Discussion

Adapting a CBPR framework for conducting this research has immensely strengthened the value and validity of the study and ultimately of its findings. For example, these methods directly resulted in the targeted recruitment plan of households in the community to be able to include and compare households both receiving and not the government cash-transfer for supporting orphaned children. Had we not consulted widely with key government officials and the community, we likely would have missed this important opportunity. Further, by working through existing mechanisms such as the UG Children’s Services Forums, we ensured that we were able to approach all organizations and facilities in the county caring for or supporting orphaned children. As a result, our study findings will ultimately be richer, and more generalizeable, than otherwise would have been the case. Similarly, it is only through leveraging and working through existing community-based structures that were able to obtain a random sample of households caring for orphaned children in the community, and retain a majority of those children, including those who are street-involved, in the study, now for 3 years. We did however face several important challenges which we describe here with the intention of informing other investigators in their use and adaptation of CBPR methods.

### Balancing community needs

One challenge with eliciting community feedback through multiple sources is determining how to balance the community needs and requests with the general objectives of a research project. From the outset at the community *mabaraza*, the project has faced a host of community needs and requests, many of which fell outside the scope and aims of the project’s focus on orphans. There are numerous public health and socioeconomic issues affecting the communities, many of which community members expressed to be priorities in addition to orphaned children. These included mentally and physically challenged children, teenaged mother and single mothers, and the elderly members of the community. While the project considered these matters to be of great importance, it was not able to address the issues raised as they would require separate studies.

Many of the households enrolled in the study face adverse economic conditions that directly impact the health and well-being of the children they care for. Additionally, many of the study’s rural locations were affected by the 2007/2008 post-election violence in Kenya, from which communities are still in the process of recovering. Community members sought to know if the children enrolled would be entitled to benefits such as education, school uniforms and fees. Furthermore, as many families are food insecure, the project has often been requested to provide food to households.

In order to address the challenge of responding to the needs and requests of the community, the project has taken the time to listen to the communities concerns, sympathized with their challenges, and attempted to direct them to other resources available in their locations for assistance when available. Open and on-going communication, understanding and CHW’s who act as the direct link between the project and the community have been fundamental in balancing community needs and requests with the scope and aims of a research project.

### Eligibility, sampling & recruitment

Reconciling differences in beliefs about how sampling and recruitment should take place was another challenge arising from the community participation. The OSCAR project explained that it would recruit its participants from different locations at the community *mabaraza*, based on random sampling of eligible households. However, the community suggested another version of ‘*purposively*’ selecting households which they thought deserved to be considered. The community members said their decision to select households would be informed by factors such as the extent of poverty, age of caregiver, number of children, and numerous other factors.

To reach resolution about the sampling strategy, the project took time in each location and discussed the merits and demerits of their proposal with a focus on the norms that govern research projects and the need to eliminate bias as much as possible so as to bring validity to the results obtained at the end of the research. To ensure participation and transparency in the sampling method, the community was involved in providing a list of all households caring for orphaned and separated children to ensure that every household with orphans and/or separated children had an opportunity to be randomly selected. This was done at each location and was coordinated by the Chiefs, Assistant Chiefs and Village Elders. By involving the community in creating the lists of eligible households it ensured that they were involved in the sampling and recruitment plan, and participating in the research process with the OSCAR team. The accuracy of the sampling lists of eligible participants provided by the community proved challenging; in some locations, the CHW’s identified sampled households only to realize that the household did not meet the eligibility criteria of the project. That is, there were no orphaned or separated children in the household. In other cases, some sampled households had been found to be single mothers while others have only elderly couples. Many of these households were put on the list by the Village Elders, the Assistant Chiefs or the Chiefs because they perceived the households as being as needy as those with orphaned children. Other sampling challenges arose when some eligible households had not been randomly selected from the lists provided by the communities. The project worked with the Village Elders and CHW’s over a period of several months to refine their lists of eligible households to ensure all – and only – eligible households were on the lists for sampling.

### Providing access to healthcare

Access to healthcare is a challenge within UG County, with only 2 public hospitals and a doctor to patient ratio of 1:10,034 [33]. During community *mabaraza*, the numbers of children in need of medical assistance was large for both orphans and non-orphans. Often members of the community would come along with their ill children seeking assistance or guidance from the project team after the meeting. The needs ranged from simple medical conditions to complicated conditions that require specialized care at tertiary health facilities. The community members understood that the project was to carry out annual health assessments on the recruited participants from the households and CCI’s and semi-annual assessments on participants recruited from the streets. Numerous questions arose in regards to providing access to healthcare due to the difficulty many families have in accessing primary care. Community members wanted to know if the study participants could visit the project clinic for free healthcare whenever they fell ill between scheduled appointments, whether the project would provide care to caregivers of the children, whether medical bills incurred by study participants seeking care at local facilities would be covered, and if the project could cover participants for specialized care, such as dentistry, ophthalmology, surgery and rehabilitation.

The requests of the community for primary healthcare were challenging to address for the project, as the line between clinical care and research were difficult to distinguish for the community. In providing additional clinical care, the project had to be conscious of the fact that the healthcare component must not be perceived to be an undue inducement for participating in the research as this would be unethical.

After meetings with the project’s co-investigators and consultations with the Moi Teaching and Referral Hospital’s Director, the team was able to find a solution to the healthcare needs of the participants while ensuring that the research remained at the forefront of the project. To meet the community’s expectations for healthcare, the project agreed to provide basic healthcare to all study participants between scheduled appointments at the project clinic, to offer free consultations to ill caregivers accompanying their children to appointments, and to provide basic pharmaceuticals at no cost for recruited participants receiving prescriptions through the clinic. Moi Teaching and Referral Hospital agreed to facilitate standard referred care to its different service points at no cost for project participants. However, specialized care or care sought outside the project clinic and MTRH would require the participant to cover the costs.

The project has faced additional clinical care challenges by receiving unscheduled visits from community members who have not been enrolled into the project but accompany recruited participants and their care givers to the clinic with the hope that they too will receive treatment. This poses an ethical challenge to clinic staff providing care and to the study team and is evaluated on a case by case basis to offer consultation and referrals. Unfortunately the study is not able to provide primary care services to non-participants. This is an on-going challenge for the study, and one inherent in doing CBPR in a resource-constrained setting.

## Conclusion

CBPR methods provide a solid and feasible framework for conducting community-based epidemiological and public health research in sub-Saharan Africa. The OSCAR’s Health and Well-being project demonstrates that community involvement and participation in the research process allows for successful implementation of a project involving a vulnerable population and within complex community structures. However, community participation and involvement in the research agenda in sub-Saharan Africa is not without challenges and requires careful consideration on the part of the research team to adequately address community opinions, needs, and level of participation in the research process. By collaborating with the community and stakeholders from research question development, through to implementation and dissemination of results, epidemiological and public health research projects in sub-Saharan Africa have the potential to increase the validity and generalizability of the research. In this manner, local communities and their civil representatives are better positioned to address public health issues important to the community, and create sustainable interventions and solutions to key issues affecting them.

## Competing interests

The authors declare they have no conflict of interests.

## Authors’ contribution

AK & LE contributed equally to the development of the manuscript. DA & PB conceived, designed and supervised the study and assisted in writing the manuscript. LA, PG, SA & RV contributed to the study design and assisted in writing in the manuscript. All authors read and approved the final manuscript.

## Pre-publication history

The pre-publication history for this paper can be accessed here:

http://www.biomedcentral.com/1471-2458/13/91/prepub
